# Major scientific lessons learned in the trauma field over the last two decades

**DOI:** 10.1371/journal.pmed.1002339

**Published:** 2017-07-05

**Authors:** John B. Holcomb

**Affiliations:** Center for Translational Injury Research, Department of Surgery, McGovern Medical School, UT Health, Houston, Texas, United States of America

## Abstract

John B Holcomb summarizes recent conceptual and practical advances in trauma care, in both military and civilian settings, and presents directions for future research.

Over the last 20 years, care for injured patients has undergone a revolution. As noted by many authors, war’s only silver lining is to improve the care of the injured, and this era is no exception. Why do wars always seem to change the existing paradigm? Most experienced military personnel describe a 100% focus on the injured, stemming from the emotional impact of proximity to the battlefield, the close living and working quarters of medical personnel and combatants, and the sense of duty towards those who are injured while serving their country. Whatever the reasons, the results speak for themselves. The present era of conflict, starting on September 11, 2001 and continuing today is no exception. Amazing changes have occurred in care in the combat theater, and some of these have transitioned into the civilian world. This is critically important, as the scope of the civilian injury problem is 300 times that of the military, while military-style injuries are, unfortunately, becoming more common in civilian life.

Trauma teams used to “stay and play”: we’d start with two large-bore intravenous (IV) lines and if the patient lived several hours infuse 20–30 liters of crystalloid and transfuse 10 units of red blood cells (RBCs) before thinking about ordering any other component. We would stay in the operating room and repair all injuries and then close the fascia on everyone, delay repair of fractures, use high tidal volume ventilation, and studiously avoid any minimally invasive techniques. The inevitable coagulopathy, renal failure, abdominal compartment syndrome, wound complications, and acute respiratory distress syndrome were considered acceptable diseases of survivorship. Death rates were high, survivors infrequently utilized rehabilitation centers, and follow-up for traumatic brain injury (TBI) and post-traumatic stress disorder (PTSD) patients was rare.

In the following few words, many areas of improvement are described. As with all descriptions of current status, the work is not done and continued progress is mandatory. Of course, this list is not all-inclusive; inevitably something important has been left out, and for that I apologize in advance.

## Training

For all members of the trauma team, training used to be haphazard, simulation centers were essentially nonexistent, and military personnel were trained utilizing the same concepts that were developed for civilian casualties. Today, prehospital and hospital team training is commonplace and integrated, simulation centers are widespread and the training and equipment designed for the military environment is commonplace as Tactical Combat Casualty Care (TCCC) spreads across the globe [1]. A focus on current trauma experience has become accepted as critical for optimal patient outcomes in both the short and long term [2]. These concepts have transitioned variably into clinical practice in the civilian world, where mass casualty and terrorist events are increasing, and the huge numbers of everyday trauma cases could benefit from hard-won military lessons. Uniform translation of these concepts into both the military and civilian sectors is unfortunately incomplete.

## Systems of care

In the early 2000s, the all-important comprehensive approach to injury, from prevention, into acute care and through rehabilitation, including performance improvement, training, registry, and research (i.e., a comprehensive trauma systems), were nonexistent on the battlefield, with physicians and medical and line commanders unaware of individual clinical outcomes or system-wide best practices. In response to this, and leveraging a well-established concept from the civilian arena, The Joint Trauma System (JTS) was created and implemented, quickly becoming the standard across all levels of combat casualty care [3]. Utilizing the concepts of a learning healthcare system, the JTS could serially improve outcomes after injury across all levels of care. Fundamental to this approach was an assessment of every death for potential preventability, which drove clinical improvements and focused research funding [4]. Unfortunately, these comprehensive trauma systems concepts are already being forgotten, as today’s battlefield is being fought without the guiding presence of the JTS.

## Stopping bleeding is important

The idea that it is important to stop bleeding sounds so obvious, but 20 years ago the emphasis when faced with bleeding patients was largely on resuscitation to various oxygen or cardiac output-based endpoints. Today, we more clearly understand that a variety of interventions are required to save lives. The relationship between the multitude of hemorrhage control devices, (truncal, junctional, extremity, intravascular, and intraperitoneal) combined with hemostatic resuscitation and rapid operative intervention, is critical for survival. This bundled approach to hemorrhage control minimizes the duration and depth of shock, while surgeons repair the endothelium and restore hemostatic competency [5]. Integration and implementation of these concepts results in decreased hemorrhagic death and edema-related complications and improves patient outcomes.

## Transfusion

Those who die after injury frequently do so from exsanguination, the leading cause of potentially preventable death. Bleeding to death occurs rapidly (within 6 hours of admission), and understanding the time course of hemorrhagic death is critical towards effective intervention. Through the conflicts of the last 20 years, we have developed the current treatment for traumatic hemorrhagic shock; simultaneous mechanical hemorrhage control coupled with damage control resuscitation (DCR), with an emphasis on using plasma as the primary resuscitative fluid [6]. DCR principles include minimization of crystalloid and artificial colloids, permissive hypotension and balanced resuscitation with early platelets, plasma, cryoprecipitate, and RBCs [7]. Optimal resuscitation now starts in the prehospital area with blood products. However, the current paradigm is changing with the TCCC guidelines recommending a move from balanced component transfusion in favor of whole blood (WB) [8]. This important transition has been led by the military with more than 10,300 units of WB transfused in the war, with favorable clinical outcomes. Several civilian trauma systems are now routinely using WB, both prehospital and in the hospital [9].

## Neurosurgery and orthopedics

Receiving a penetrating brain injury was previously assumed to be fatal and the idea of aggressive intervention by medical personnel misplaced. However, the experience gained through treating a large number of penetrating brain injuries in the current conflicts has reconfirmed the utility of aggressive surgical intervention and maximal intensive care [10]. Future randomized studies focused on these issues are warranted, especially as civilian injuries become more similar to those experienced by military personnel. However, the true signature wounds of war are those of the extremities, with thousands of fractures managed and soft tissue debridement’s performed [11]. We learned early that low-pressure and high-volume irrigation combined with serial debridements resulted in the cleanest wounds, and that transport with negative pressure devices was safe and greatly facilitated wound care [12–14].

## Research funding

Injury is the leading cause of life years lost and the number one cause of death in people under 47. Despite this well-known fact, research funding lags far behind every other disease [15]. Continuing this abysmal level of funding by our elected and appointed officials ensures that the societal damage inflicted by this disease will continue unabated. During intervals of relative peace, advances in trauma care usually occur in the civilian arena and are driven by injury research funding. The recent National Academy of Medicine report specifically highlights this 50-year disparity in research funding and its continued impact on the health of all nations [16]. Lack of this funding essentially guarantees lower levels of preparedness at the start of the next war [17].

## The future

What does the future look like? While impossible to predict with any certainty, I believe that advances will continue in prehospital resuscitation and hemorrhage control, extending the survivable prehospital and time to operative intervention. We must decrease mortality and morbidity from sepsis, all types of TBI, and improve pain control and outcomes from PTSD. Successful rehabilitation after all injury and reintegration into the workforce must become a focus for every trauma patient. Cellular therapy will become an important early intervention to appropriately modulate the inflammatory system, decreasing multiple organ failure and rebuild or replace damaged organs [18].

The most important advance, however, lies within the realm of leadership. Our military and civilian leaders must implement the lessons learned on the current battlefield and be held responsible for clinical outcomes across all levels of care, wherever the injury occurs [19–21].

## Abbreviations

DCR: damage control resuscitation
IV: intravenous
JTS: Joint Trauma System
PTSD: post-traumatic stress disorder
RBC: red blood cell
TBI: traumatic brain injury
TCCC: Tactical Combat Casualty Care
WB: whole blood
